# Geographic correlation between deprivation and risk of meningococcal disease: an ecological study

**DOI:** 10.1186/1471-2458-4-30

**Published:** 2004-07-26

**Authors:** Christopher J Williams, Lorna J Willocks, Iain R Lake, Paul R Hunter

**Affiliations:** 1 East & North Hertfordshire Health Protection Unit, Welwyn Garden City, Hertfordshire AL8 6JL, United Kingdom; 2 Health Protection Agency East of England, Cambridge CB2 2SR, United Kingdom; 3 School of Environmental Sciences, University of East Anglia, Norwich, United Kingdom; 4 School of Medicine, Health Policy and Practice, University of East Anglia, Norwich, United Kingdom

**Keywords:** Meningitis, meningococcal, Meningococcal infections, Communicable disease, Geography, Maps, Socioeconomic factors.

## Abstract

**Background:**

Meningitis caused by *Neisseria meningitidis *is a serious infection which is most common in young children and adolescents. This study investigated the relationships between the incidence and age distribution of meningococcal disease, and socioeconomic environment.

**Methods:**

An ecological design was used, including mapping using a Geographical Information System (GIS) at census ward level.

**Results:**

Incidence of meningococcal disease was highest in the most deprived wards, with a relative risk of 1.97 (1.55 – 2.51). Mapping revealed geographical coincidence of deprivation and meningococcal disease, particularly in urban areas. Two-thirds of the increased incidence was due to cases in the under fives.

**Conclusions:**

The results suggest that area deprivation is a risk factor for meningococcal disease, and that its effects are seen most in young children.

## Background

Infections caused by *Neisseria meningitidis *are still an important cause of morbidity and mortality in the United Kingdom, with 1548 cases notified in England and Wales in 2001[1], an incidence rate of 3.2 cases per 100,000 per year, and a case fatality rate of around 10%[2].

The infection is most common in infants and adolescents, with peaks of incidence at 0–5 years and 15–19 years[2]. Outbreaks cause high degrees of anxiety in local populations, though 95% of cases are sporadic[2]. It is not clear how the bacterium spreads through the population in time and space, or what determinants are most important in each area. Suggested environmental risk factors for meningococcal infection include passive smoking[3], overcrowding[4], and weather conditions[5,6].

It is accepted that many infectious diseases preferentially affect the most disadvantaged in society. This has been discussed at historical[7], global[8], and national[9] levels, but new geomapping techniques have shown that it holds true when comparing small areas such as postal or electoral districts. The environmental and social factors conferred upon an individual through their residence in a particular area may be as important as the individual 'risk factors' where communicable disease is concerned[10]. Mapping of socioeconomic status and certain infectious diseases, in particular sexually transmitted infections [10-13], has shown a relationship between area socioeconomic indicators and disease incidence. Geographical Information Systems (GIS) techniques are able to both test hypotheses about the geographical distribution of disease and to display environmental characteristics and disease incidence in a clear and interpretable way.

This study was prompted by an impression, gained from the practice of a consultant in communicable disease control (CCDC), that infant cases of meningococcal disease came from families of low socioeconomic status, whilst older cases tended to be drawn from families of higher socioeconomic status. Studies in northeast Thames[14], southwest England[15], and Wales[16] have shown an increase risk of meningococcal disease in more deprived areas, particularly in the under five age group. This study aims to test these hypotheses concerning age, area socioeconomic status and meningococcal incidence, using a larger sample size and a Geographical Information System (GIS). GIS methods are used to display the information on deprivation and disease incidence in an informative way, enabling the viewer to formulate new hypotheses about disease transmission in the region.

## Methods

The study was ecological in design, and used census-derived area data, map data, and individual case data as described below. The geographical unit of analysis was the 1991 census ward, and the study population was the entire Eastern region population of the UK (1999 estimate 5.3 million).

The Eastern region of England is made up from the counties of Hertfordshire and Essex (both adjoining Greater London to the south), Suffolk and Norfolk along the east coast, Cambridgeshire centrally and Bedfordshire to the north. Much of the area is rural, although there are several medium-sized urban areas in Essex and Hertfordshire, with links to the capital. Away from the London area, the major urban centres include Cambridge and Norwich (both with large university populations), the ports of Ipswich and Harwich, and Peterborough and Luton.

### Data sources

Data on cases came from the Eastern region Communicable Disease Surveillance Centre (CDSC Eastern) regional database of enhanced surveillance of meningococcal disease[17]. To be included in the surveillance data, a case had to fit the Public Health Laboratory Service (PHLS) case definitions[18] at a local level, and also had to have been included in the monthly returns sent to the regional level. The study used full postcode and age band information on all cases from 1999 and 2000, a subset of the enhanced surveillance data. Linked information on cases, such as the serogroup or date of notification, was not requested for this study. This also meant that clusters could not be excluded from the analysis.

The regional population data were projections for 1999 based on 1991 census data, obtained from the compendium of clinical and health indicators 2001[19]. This source gave age-banded population data at 'synthetic ward' level (single or aggregated census wards, producing a population of over 5000). Each synthetic ward population was shared equally between all its constituent census wards.

The deprivation index used was the Townsend score, which combines local measures of unemployment, car ownership, overcrowding, and housing tenure[20]. This measure was used as it has already been widely used in similar literature[11,14,15,21], does not include potential confounders such as percentage of under fives, and was available at census ward 1991 level. A higher Townsend score indicates a more deprived area. Ward level scores based on 1991 census data were obtained from the Manchester Information and Associated Services (MIMAS)[22].

The shape files used in the MapInfo (© MapInfo Corporation) and EpiMap2000 (in Epi Info™, Centres for Disease Control and Prevention) maps were digitised ward boundaries from the 1991 census, obtained from the EDINA (Edinburgh data and information access) UKBORDERS service[23]. Vector-based files of census ward boundaries in the counties of Bedfordshire, Cambridgeshire, Essex, Hertfordshire, Norfolk and Suffolk, which make up the UK Eastern region, were downloaded in combined form. Ward population density was calculated using the ward areas contained in the map files, and the population estimates described above.

### Ethical approval

Ethical approval was obtained from the West Hertfordshire health authority local research ethics committee. An application form was submitted, along with a copy of the project protocol, and written approval was returned.

### Analyses

Cases were mapped to census wards using their postcode georeference (NHS postcode database). Microsoft Access was used to assign the deprivation scores, to divide and manipulate ward data, and to link the geographical and attribute files. The age distribution of cases and incidences was calculated using Microsoft Excel, which was also used to create the charts.

Statistical testing was performed using StatsDirect (^©^StatsDirect Ltd), apart from the relative risk confidence intervals, which were calculated using Microsoft Excel[24]. Poisson confidence intervals for ward and deprivation group incidence rates were calculated by using the number of cases and the total person-years at risk (twice the ward or deprivation group population). Chi-squared tests for trend were performed for the successive incidence rates across the deprivation groups. The variation in the age distributions of incidence and case counts was compared using the non-parametric Friedman and Mann-Whitney tests respectively. Two Poisson regression models was constructed using StatsDirect, with incidence as the dependent variable and Townsend score and population density, and Townsend score alone, as predictors.

The maps were produced with MapInfo, using the "range" function to colour each ward in shades depending on the level of deprivation or incidence of disease. The superimposed maps used a magnified version of the deprivation map in figure 3, and a stick figure to represent the magnitude of ward disease incidence.

## Results

### Case data

A total of 773 cases were reported to the CDSC (Eastern) enhanced meningococcal database during 1999 and 2000. Of these, 524 had some postcode details and 499 had the full postcode. 458 cases had a ward assigned, and, of these, 451 had a Townsend score. These data losses were due to postcodes not being recorded (the major factor) or incorrect, discrepancies within the NHS postcode file, and incomplete ward deprivation data (this only relates to the 524 – 451 cases, not the bulk 773-524 cases). Further analysis is therefore restricted to the 451 cases with Townsend score. Where incidence rates are given, they will generally be underestimates of the true incidence (on average 58% of the true value), due to the loss of case data.

**Figure 1:**
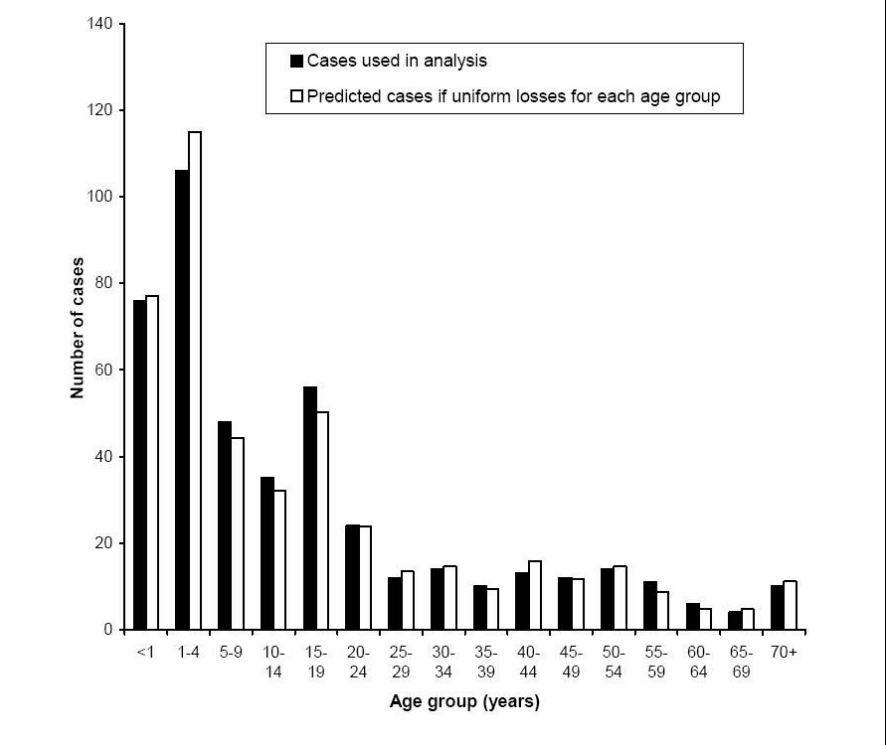
Case losses*: Comparison of numbers of cases included in analysis, by age group, with expected values if losses were equal across all age bands *Sources *Cases: *Confirmed and probable cases of invasive meningococcal disease included in analysis, enhanced surveillance data from CDSC eastern

It was assumed that the data losses were random with respect to the variables of interest. The age structure was well preserved despite the loss of around 40% of the initial cases. Figure 1 compares cases included in the study (451) with the numbers expected if losses were uniform across the age groups. Chi squared testing confirmed that the losses were not related to age group (P = 0.8529). When broken down into the eight Health Authorities supplying case data, the percentage of cases that included postcode information was roughly similar, with around 2/3 of cases being postcoded, with the exception of one authority where only 38.6% of cases were postcoded. However, this authority only contributed 7% of the cases.

Of the 1184 wards included in the analysis, 325 (27%) had at least one case of meningococcal disease in the two-year period. In these wards, the maximum number of cases was 6, and the median 1.

### Incidence of meningococcal disease

The overall incidence for 1999 and 2000 was 7.4 cases per 100,000 per year. Within the wards containing more than one case, the median incidence was 12.9 per 100,000 per year, and ranged from 3.7 to 60.0. Given the small numbers involved, Poisson confidence intervals for these incidences are wide. In a high incidence ward (41.7 cases per 100,0000 per year), the confidence interval was 11.4 to 106.8 cases per 100,000 per year. In a low incidence ward (4.0 cases per 100,000 per year), the confidence interval was from 0.1 to 22.5 cases per 100,000 per year. The ward incidences will be underestimates due to the loss of cases described above.

Figure 1 also shows the age distribution of the cases. This bimodal distribution shows that the peak incidence is in children under 5, with a second peak in the 15–19 group.

**Figure 2:**
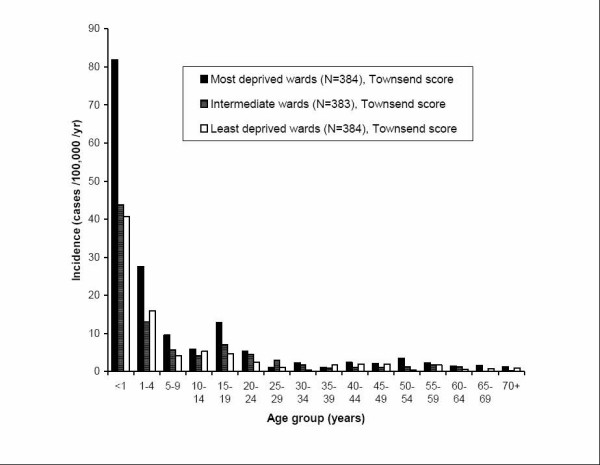
Age-specific incidence rates*: Comparison of cases in each Townsend score group (thirds, 1151 wards) *Sources *Cases*: Confirmed and probable cases of invasive meningococcal disease included in analysis, enhanced surveillance data from CDSC eastern *Denominator*: 1999 population estimates, compendium of clinical and health indicators 2001, adjusted from synthetic wards (see methods) *Map files *: 1991 Census digitised boundary data *Townsend scores*: 1991 Census area statistics

### Incidence and deprivation

Table 1 shows the summary figures for the wards analysed. The mean incidence of meningococcal disease in the most deprived wards is twice that in the least deprived wards (5.9 versus 3.0), with a relative risk of 1.97 (1.55 – 2.51). The intermediate wards have an overall incidence similar to that in the least deprived wards. A chi-squared test for trend shows a significant variation between the rates in each group (chi squared for linear trend = 39.0, p < 0.0001).

**Table 1:** Meningococcal disease in the eastern region, 1999 and 2000: Incidence rates after division into thirds by ward Townsend score

	***Least deprived wards***	***Intermediate wards***	***Most deprived wards***
**Number of wards ^a^**	384	383	384
***Range of ward Townsend scores***	-6.26 to -2.19	-2.2 to-0.33	0.34 to 8.44
***Number of cases ^*b *^(all ages)***	88	97	267
***Total population ^*c *^(all ages)***	1,473,272	1,506,359	2,271,294
***Incidence [corrected] ^*d*^***(cases /100,000 /yr)	3.0 [5.1] (2.4 – 3.7)^*e*^	3.2 [5.5] (2.6 – 3.9)	5.9 [10.1) (5.2 – 6.6)
***Incidence relative risk compared to least deprived wards [CI]***	**1.00**	**1.07 **[0.80–1.43]	**1.97 **[1.55–2.51]

(a) Census wards 1991, divided by Townsend index, from 1991 census (b) All cases of meningococcal disease (confirmed and probable) from enhanced surveillance data, CDSC eastern, included in analysis (c) Population estimates for 1999, Compendium of Health and Clinical indicators 2001, adjusted as in methods (d) Corrected to allow for the loss of 42% of cases. Corrected incidence = uncorrected/0.583. Assumes losses are equal across deprivation groups (e) Poisson confidence intervals – see methods

Poisson regression using population density and Townsend score as predictors revealed population density to be a non-significant contributor to the variation in incidence (p = 0.086). A second model, setting incidence against Townsend score alone, suggested that ward incidence rises by 12% (9 – 16%) for every unit increase in deprivation score.

### Age distribution and deprivation

The age-specific incidence rates are shown in figure 2. The most striking feature is the large excess of cases in the under ones and one to fours in the most deprived wards. The incidence is 1.9 times higher for the most deprived under fives (under one and one to four groups combined). The increased incidence in the under five age group accounts for 68% of the difference in overall incidence between the most and least deprived wards. The incidences in the 16 age groups used varied significantly between the three deprivation groups (Friedman test, p < 0.0001).

**Figure 3:**
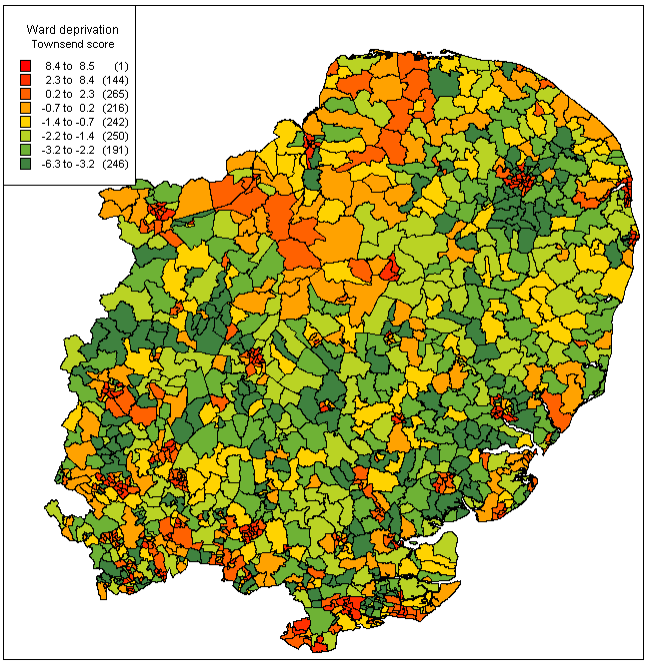
Ward deprivation in the Eastern region, by Townsend score* *Sources *Map files *: 1991 Census digitised boundary data *Townsend scores: *1991 Census area statistics *Software*: MapInfo^© ^Professional

The Poisson confidence intervals for these incidences are narrower than for individual wards. Two were calculated: 21.3 to 35.1 for an incidence of 27.6 per 100,000 per year, and 0.3 to 4.9 for a lower incidence of 1.7 per 100,000 per year.

### Mapping deprivation and meningococcal disease

Figure 3 shows ward deprivation by Townsend score using MapInfo. The wards are divided into eight bands by their ward score. Many of the most deprived wards correspond to urban areas, though there was a broad area of greater deprivation in the north of the region.

**Figure 4:**
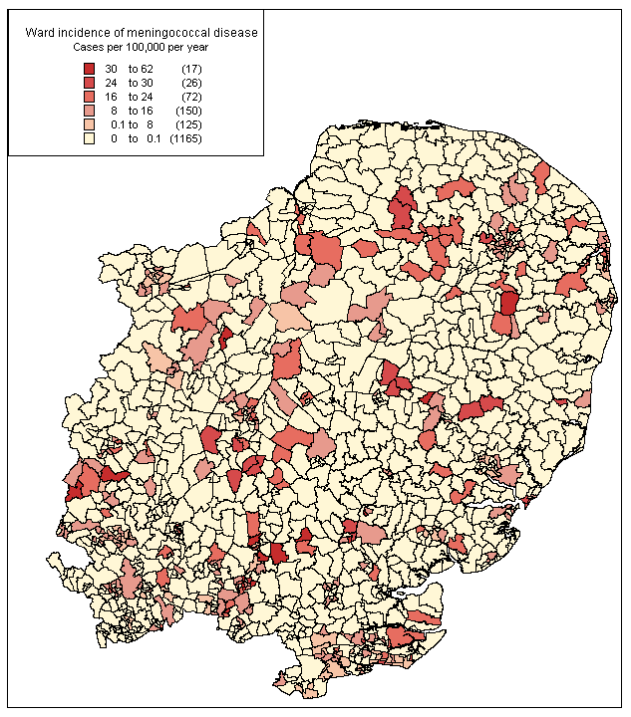
Eastern Region, 1999 and 2000: Incidence of meningococcal disease by census ward 1991* *Sources *Cases*: All cases of meningococcal disease (confirmed and probable) collected by CDSC Eastern for enhanced surveillance & included in analysis, 1999 and 2000 *Denominator*: 1999 population estimates from Compendium of Health and Clinical Indicators 2000, adjusted for true ward (see methods) *Map files *: 1991 Census digitised boundary data *Software*: MapInfo^© ^Professional

Figure 4 shows a map of meningococcal incidence by ward in the region, with colours coded by six incidence ranges. Areas of high incidence often coincide with urban regions, though some rural areas also have high rates of disease. These include some of the deprived rural areas, including the area in north Norfolk noted above.

**Figure 5:**
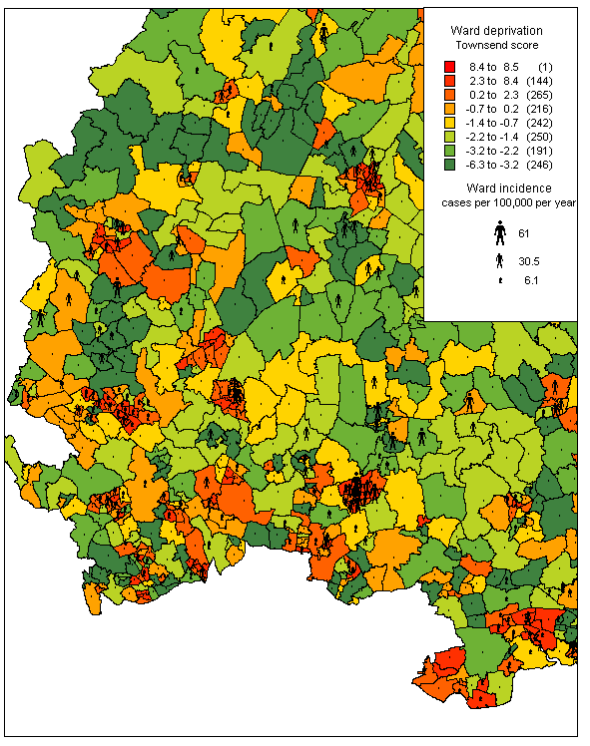
Meningococcal incidence and deprivation superimposed, Hertfordshire and west Essex* *Sources *Map files *: 1991 Census digitised boundary data *Townsend scores*: 1991 Census area statistics *Software*: MapInfo^© ^professional *Cases*: confirmed and probable cases of invasive meningococcal disease included in analysis, enhanced surveillance data from CDSC eastern *Denominator*: 1999 population estimates, compendium of clinical and health indicators 2001, adjusted for true ward (see methods)

Figure 5 shows incidence rates superimposed on the deprivation map from figure 3, and magnified to show local detail in the Hertfordshire/Essex region. It shows the relationship of incidence to deprivation, with high incidence wards being clustered within and around the 'foci' of deprivation. This is particularly marked in Harlow, represented by the cluster in the lower central part of the map.

## Discussion

The results for the regional surveillance data support the theory that meningococcal disease is associated with socioeconomic deprivation. Compared to the Welsh study[16] this study included more cases (451 vs. 295) over a wider area, with a total population of 5.3 million. Also, both deprivation and incidence of meningococcal disease are mapped using GIS, showing the spatial relationship of disease foci to areas of deprivation, both urban and rural. The study, in common with that from southwest England[15], also shows that this relationship holds in an area containing many rural wards, despite the problems associated with deprivation indices in rural areas[25,26].

The analyses of the age distribution of cases and incidences suggest that there is variation between the deprivation groups. There was a significant difference between the age-specific incidence rates between the groups, and a median age difference of five years between the most and least deprived groups (non-significant). The relative risk of disease in the most deprived wards compared to the least deprived (1.97, CI 1.55 – 2.51), is greater than that observed in the northeast Thames study[14] (odds ratio 1.51 for N. meningitidis), and the southwest England study[15] (relative risk 1.76, between upper and lower quartiles), but less than that found in the Welsh study[16] (relative risk 2.4 between upper and lower quintiles). The incidence of other infections, such as infectious intestinal disease, has also been shown to vary with area socioeconomic conditions (relative risk 2.41, quintiles) [27].

All but two (25–29 years, 35–39 years) age groups experienced higher age-specific rates in the most deprived group, but the highest case numbers were seen in the under 5 age group. The increased incidence in the under fives in the most deprived group, compared to the least deprived, accounted for 68% of the total difference in age-specific incidence. This suggests that children under five are more vulnerable to meningococcal disease in the most deprived areas. The continuation of a similar pattern when case counts, percentage and age-specific incidence were considered improves the robustness of this conclusion.

Several environmental factors might contribute to the increased risk of childhood meningococcal disease in more deprived areas. Childcare arrangements, necessitated by either personal or environmental circumstances, might expose a child in a more deprived area to more potential carriers. Smoking is also known to be more common in people from disadvantaged backgrounds and a number of studies have identified passive smoking as risk factors for both nasopharyngeal carriage and meningococcal disease [3,28-30].

An area with a relatively greater number of under fives might have a higher overall incidence rate, simply because there are more susceptible individuals. Analysis of the percentage age distribution of the populations in each deprivation group does not reveal any marked variations. There is a slight excess of 20–29 year olds in the most deprived wards compared to the other wards, but not an excess of under fives.

Mapping of the incidence rates and deprivation indices by ward definitely added value to the routine surveillance data, emphasising the focal nature of disease, and the relationship of these foci to areas of higher deprivation. Mapping incidence at ward level, rather than pinpointing individual cases as in the Welsh study[16], both considers the population at risk, and avoids potential problems with case confidentiality. The maps also suggest that, in the Eastern region, invasive meningococcal disease is largely an urban problem. Although the higher population density of urban areas might explain this, the regression model suggested that the variation was better explained by ward deprivation (term for population density non-significant, p = 0.0866). Some of the highest rates were seen in deprived urban parts of Essex. Several rural wards containing cases corresponded to more deprived areas, particularly in North Norfolk. Figure 5 shows the relationship of incidence to deprivation in close-up, and also that disease is not confined to the more deprived areas.

This is an ecological study, so the associations shown may not be valid at an individual level. Population density might still be a factor, as the regression is subject to some autocorrelation, the population variable occurring in the incidence (dependent) and population density (predictor) terms. A separate analysis, of urban and rural areas, might show whether this is the case. The paucity of cases in rural areas might make this difficult unless several more years' data were included. The north east Thames study[14] did, however, show the same relationship in an area of high population density, as have other urban studies of pertussis[31] and sexually transmitted infections[12,21].

Geographical bias in postcoded data might have contributed toward the results, as the health authority with the lowest rate of postcode inclusion, and therefore the greatest loss of data, includes many of the more affluent parts of the region. The cases supplied by this authority only accounted for 7.4% of all cases and 4.4% of postcoded cases, so this potential bias is unlikely to have had a major effect.

Routine childhood immunisation with meningitis C conjugate vaccine started in November 1999[2], so should have had an effect on the year 2000 cases. The subset of enhanced surveillance data used for this study did not include the date of notification or the serogroup involved, so no comment can be made on the influence of the vaccine or of serogroup on the results in this paper. A breakdown of the serogroups was available for the years as a whole; of those where the group was known, 60% of cases were group B and 33% group C. Patterns of transmission may change as group C infections decline, and this might be seen with routine mapping of case data.

## Conclusions

Mapping of deprivation indices and meningococcal cases is a useful tool in the analysis of routine surveillance data. Mapping of incidence rates revealed an association between areas of high incidence and areas of higher deprivation by Townsend score. High incidence and deprivation often coincided in urban areas. Mapping of deprivation indices also reveals areas of rural deprivation, such as the coastal band in north Norfolk.

Yearly mapping of routine surveillance data can help to target control strategies for meningococcal disease locally. Analytic studies would be helpful in elucidating the mechanisms by which socioeconomic conditions influence the risk of meningococcal disease in the region. Along with the study described here, knowledge gained from such investigations could inform the work on health inequalities, and try to reduce such inequalities through health promotion and community infection control.

## Competing interests

None declared.

## Authors' contributions

CJW obtained and analysed the case and geographical data, performed the statistical analyses and mapping, and wrote the text of the paper. PH suggested the idea for the study and helped develop the methods by which the question was addressed. LW provided the data from the enhanced meningococcal surveillance database and advised on data quality and sources. IL provided expertise in geographical information systems and postcode geography.

LW, PH and IL all advised on the design of the study, and the analysis and interpretation of the results. All authors contributed to drafting the paper and have read and approved the final draft.

## Pre-publication history

The pre-publication history for this paper can be accessed here:


